# Colony and population genetic structure of the newly invasive white‐footed ant (*Technomyrmex difficilis*) in the United States

**DOI:** 10.1111/1744-7917.70196

**Published:** 2025-11-12

**Authors:** Kuan‐Ling Kelly Liu, Pierre‐Andre Eyer, Anjel M. Helms, Robert T. Puckett, Edward L. Vargo

**Affiliations:** ^1^ Department of Entomology Texas A&M University College Station Texas United States

**Keywords:** aggression behavior, breeding structure, CHC, COI, microsatellite, nestmate recognition

## Abstract

The invasive white‐footed ant *Technomyrmex difficilis* has emerged as a rising pest in several regions, yet its invasion dynamics remain underexplored. This species outcompetes native ants and causes agricultural losses by tending pest insects, including aphids and mealybugs. This study provides the first integrated analysis of the species’ behavioral, chemical, and genetic variation across Texas and Florida populations. Observations suggest that the recently discovered Texas population of white‐footed ants originated from Florida. Microsatellite and mitochondrial DNA analyses revealed low genetic diversity in both populations, with a shared haplotype consistent with the Texas population originating from Florida. STRUCTURE analysis further supported genetic clustering between the two regions. Despite similar within‐colony coefficients of relatedness for workers, the populations differed in reproductive strategy: Florida colonies showed signs of inbreeding and high inter‐colony aggression, whereas Texas colonies exhibited potential localized outbreeding, low aggression, and more uniform cuticular hydrocarbon profiles. Aggression was positively correlated with chemical divergence but not with genetic differentiation. This study establishes a foundational understanding and highlights the importance of integrating multiple types of data to understand the invasion biology of *Technomyrmex difficilis*.

## Introduction

Invasive species are frequently introduced through anthropogenic activities and have the ability to establish and proliferate in novel environments where they can have significant ecological, economic, and public health impacts. The ecological impacts of invasive species are well‐documented, including disruptions to native species populations, alterations in community structure, and extinctions (Pyšek *et al.*, 2020; Bellard *et al.*, 2021). In the United States alone, the economic impact of invasive species is estimated to cost $120 billion annually (Pimentel *et al.*, 2005). In addition, some invasive species can pose serious public health risks by acting as vectors for diseases, exacerbating allergies, or causing physical harm (Jemal & Hugh‐Jones, 1993; Wilke *et al.*, 2020).

Globalization has accelerated the spread of invasive species, often along trade routes, contributing to widespread ecological and economic damage (Simberloff, 2013; Bertelsmeier, 2021). A key driver of this spread is the “bridgehead effect”, where an initially introduced population serves as the source for further invasions (Lombaert *et al.*, 2010). Several studies have documented this pattern in invasions of social insects, revealing the importance of secondary introductions in their global expansion (Ascunce *et al.*, 2011; Bertelsmeier *et al.*, 2018; Blumenfeld & Vargo, 2020; Blumenfeld *et al.*, 2021). Specifically, Bertelsmeier *et al.* (2018) found that invasive ants are more frequently exported from other introduced regions than from their native range.

In addition to dispersal mechanisms like the bridgehead effect, the social structure of invasive ants, particularly those displaying unicoloniality, plays a key role in their ecological dominance and range expansion. Unicoloniality is a highly cooperative social structure exhibited by some invasive ant species, where numerous nests coexist without aggression, forming expansive networks of workers, brood, and reproductive queens (Holway *et al.*, 1998; Holway *et al.*, 2002; Tsutsui & Suarez, 2003). This absence of intraspecific territoriality is linked to the high similarity of chemical recognition cues, primarily cuticular hydrocarbons (CHCs), across colonies, which reduces workers’ ability to distinguish nestmates from non‐nestmates (Vander Meer & Morel, 1998). This shared “colony odor” is established and maintained via trophallaxis, allogrooming, and direct contact (Bradshaw & Howse, 1984; Liu *et al.*, 2000). The degree of variation in CHC profiles between nests can influence aggression levels, with greater differences generally leading to increased aggression, thereby affecting colony boundaries and cooperative interactions. However, behavioral assays performed by researchers alone may not fully capture the social structure, as ants can sometimes recognize non‐nestmates without displaying overt aggression (Breed, 2003). Given these complexities, integrating alternative methods such as genetic and CHC profile analyses can be crucial for assessing intraspecific interactions.

The white‐footed ant, *Technomyrmex difficilis* Forel, 1892, is native to Madagascar (Wetterer, 2013) and recently invaded the United States, likely from the transport of infested residential landscaping plants and materials (Warner *et al.*, 2002). Until Bolton (2007) clarified its taxonomy, this species was often misidentified as *T. albipes* (F. Smith, 1861) in both field and collection records, with several populations worldwide, including some in Florida and the West Indies, likely representing *T. difficilis* rather than *T. albipes* (Wetterer, 2008). This revision has important implications for understanding the species’ true distribution and invasion history, and should be considered when interpreting historical records of its spread.

In the United States, *T. difficilis* was first found in Miami‐Dade County, Florida in 1986 and have now expanded their invasive distribution, establishing colonies in 22 nearby counties, and several states (Georgia, North Carolina, South Carolina, Louisiana; Warner *et al.*, 2002; Warner & Scheffrahn, 2004). Colonies of *T. difficilis* can become quite large, containing millions of individuals and may include both ergatoid (wingless) queens and males, along with the typical alate (winged) sexual forms (Tsuji & Yamauchi, 1994; Warner, 2003; Bolton, 2007), creating a nuisance to homeowners as they forage both inside and outside of buildings. They are also an agricultural pest because they tend mealybugs and aphids, shielding them from natural enemies and exacerbating crop damage (Samways *et al.*, 1982; Sulaiman, 1997). *Technomyrmex difficilis* has become established as a dominant species in southeastern Florida, invading intact forest habitats and impacting native species (Wetterer, 2013; Wetterer, 2018). In 2019, white‐footed ants were found for the first time in Texas (Corpus Christi; Robert Puckett, personal communication). The species was discovered inhabiting the pot of a palm tree originating from Florida and subsequently dispersed within the grounds of the Texas State Aquarium. Although no additional Texas populations of *T. difficilis* have yet been identified, it is important to understand the origin and ecology of this species to control existing populations and prevent future introductions.

Beyond the US mainland, *T. difficilis* has demonstrated a broader invasive potential and ecological impact in other regions. In Hawaii, Tong (2014) found *T. difficilis* to be the most common *Technomyrmex* species and the third most frequently encountered ant overall, present in 27% of surveyed sites across diverse vegetation zones. Its widespread presence and association with higher ant densities suggest rapid expansion, likely driven by low interspecific competition and recent introduction. In the West Indies, its high densities and competitive dominance have likely contributed to the displacement of native species (Wetterer *et al.*, 2018). In Europe, *T. difficilis* was first reported in 2015 in a greenhouse in Lyon, France. Despite being confined initially to a single building, the population was well‐established and posed a risk of spread to other locations, particularly through the movement of potted plants (Blatrix *et al.*, 2018). A similar situation has been observed in our study, where the white‐footed ant was initially found only within the Texas State Aquarium in Corpus Christi, Texas, USA. However, as of the time of this writing, the species has begun spreading to nearby buildings (Robert Puckett, personal communication).

Despite its growing distribution and ecological prominence, relatively little is known about the colony structure, aggression behavior, and invasion pathways of *T. difficilis*. A previous study suggested possible unicoloniality and low aggression levels in Florida populations (Sollins, 2010), but the consistency of these traits across regions and their relationship to colony boundaries remain unclear. The goal of this study was to compare genetic variation, aggression levels, chemical profile, and breeding structure between *T. difficilis* populations found in Florida (where they were initially identified in the United States) and the recently discovered population in Texas, which was introduced via potted plants originating from Florida. Given this known introduction pathway, we aimed to confirm the Florida origin of the Texas population and assess whether genetic, chemical, and behavioral traits have diverged since the introduction. Specifically, we tested the following hypotheses: (1) Florida populations exhibit higher genetic diversity than the Texas population; (2) the Texas population shares genetic traits such as same alleles and haplotypes with Florida populations, supporting the Florida origin; and (3) workers from different nests recognize each other as colony‐mates within the Texas and Florida populations, indicating weak nest boundaries. Overall, this study highlights the value of integrating genetic, behavioral, and chemical analyses to reveal complex patterns of colony structure and invasion dynamics in *T. difficilis*.

## Materials and methods

### Sampling and study sites

**Fig. 1:**
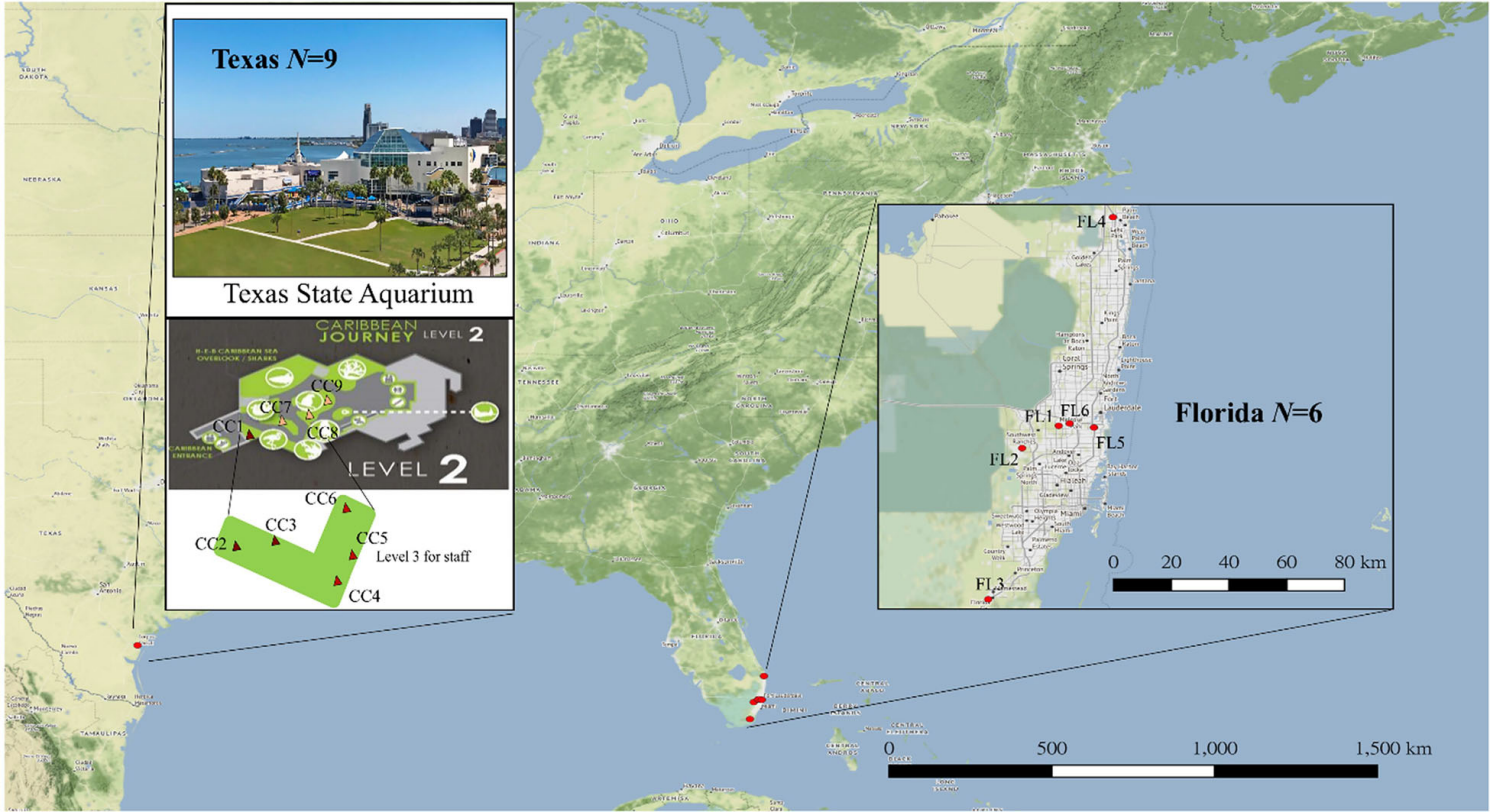
Map of the 15 collection sites which including nine nests in Corpus Christi, Texas (Texas state aquarium) and six nests in the southern part of Florida.

Nests of *T. difficilis* were collected from Corpus Christi, Texas and southern Florida (Table 1, Fig. 1). In Corpus Christi, nine nests were collected inside the Texas State Aquarium (the first infestation site reported in Texas). In Florida, we searched the southeastern coast for nests spanning from Juniper to Key West. Our search was guided by 15 occurrence records from iNaturalist (https://www.inaturalist.org/) (December 2016 to March 2022) and additional reports from the literature (Wetterer, 2008; Sollins, 2010; Wetterer, 2013). We systematically searched various habitats, including university campuses, parks, and residential neighborhoods near parks, targeting microhabitats such as tree holes, leaf litter, tree bark, and palm fronds. At each site, we spent a minimum of 20 min actively searching for nests. This survey was conducted for a full week, with intensive sampling efforts across various locations. Nests were collected from six different sites between Florida City and Palm Beach, at least 1 km away from each other (Fig. 1). We collected approximately 50 workers from each nest: eight workers were placed in a −20 °C freezer for chemical analysis and eight workers were preserved in 95% ethanol for DNA extraction. The remaining ants were kept alive for behavioral trials.

**Table 1:** Geographic coordinates of the *Technomyrmex difficilis* collection sites in Texas (TX) and Florida (FL)

ID	Location	Coordinates
CC1‐9	Corpus Christi, TX	27.813877, −97.392836
FL1	Davie, FL	26.076201, −80.272448
FL2	Silver Lakes, FL	25.994595, −80.386224
FL3	Florida City, FL	25.442797, −80.491668
FL4	Palm Beach Gardens, FL	26.831724, −80.103669
FL5	Dania Beach, FL	26.069897, −80.162757
FL6	Davie, FL	26.084517, −80.238419

### Molecular techniques

The genomic DNA of eight workers from each colony was extracted using a modification of the Gentra Puregene® kit (Qiagen, Germantown, MD, USA), in which an additional step was introduced by adding 100 *µ*L of 100% ethanol to precipitate DNA from the solution prior to the 70% ethanol wash step, and stored at 4 °C for future genetic analyses. To identify informative microsatellite markers for *T. difficilis* we evaluated 31 markers previously demonstrated to amplify in closely related species (*Linepithema humile* (Mayr, 1868), *Leptomyrmex pallens* (F. Smith, 1861), and *Tapinoma melanocephalum* (Fabricius, 1793)) (Krieger & Keller, 1999; Berman *et al.*, 2014; Butler *et al.*, 2014; Zheng *et al.*, 2018). Out of the 31 markers evaluated, 19 were eliminated due to either nonamplification or monomorphic amplification (Table S1). Twelve markers were employed for genotyping. All forward primers were 5ʹ‐tailed with an M13 sequence (5ʹ‐CACGACGTTGTAAAACGAC‐3ʹ) and a fluorescent label of 6‐FAM, VIC, PET, or NED (Boutin‐Ganache *et al.*, 2001). The Bio‐Rad thermocycler T100 (Bio‐Rad, Pleasanton, CA, USA) was used to amplify the amplicons. The PCR products were visualized on an ABI 3500 capillary sequencer and sized against a LIZ500 internal standard (Applied Biosystems, Foster City, CA, USA). Alleles were scored using Geneious v.9.1 (Biomatters, Auckland, New Zealand; Kearse *et al.*, 2012).

Sequencing of the cytochrome oxidase I gene (COI), commonly used in DNA barcoding, was performed using at least two workers from each nest. Sequences were amplified using the primers *LepF1* and *LepR1* to target a 658 base pair fragment (Hebert *et al.*, 2004; Hajibabaei *et al.*, 2006). The PCR products were purified using the EXOSAP‐it PCR purification kit (Affymetrix Inc., CA, USA) and subjected to sequencing using the ABI BigDye Terminator v.3.1 Cycle Sequencing Kit on an ABI 3500 Genetic Analyzer (Applied Biosystems). Base calling and sequence reconciliation were conducted using MEGA v.7.0 (Kumar *et al.*, 2016).

### Genetic analysis

MICRO‐CHECKER v2.2.3 (Van Oosterhout *et al.*, 2004) was used to assess the presence of null alleles and determine the suitability of microsatellite markers for the dataset. Null alleles arise from mutations in the primer binding site, preventing PCR amplification of the microsatellite locus and potentially leading to an overestimation of homozygosity. To determine whether workers collected from different nests belonged to the same colony, a log‐likelihood *G*‐test was performed between each pair of nests sampled in each state to test for genotypic differentiation using Genepop v.4.7.5 (web version; Rousset, 2008). The results of this analysis were used to assess colony structure and identify whether nests represented independent colonies. FSTAT (Goudet, 2001) was used to evaluate *F*‐statistics (Weir & Cockerham, 1984), allele frequency, the number of alleles (*N_A_*), expected heterozygosity (*H_E_*), observed heterozygosity (*H_O_*), and allelic richness (*Ar*) for each locus, using all samples per colony in each population. Colonies were treated as subpopulations in the analysis. To investigate the population structure, the Bayesian‐clustering method based program STRUCTURE v.2.3.4 (Pritchard *et al.*, 2000) was used. All samples were compared under an admixture model using Markov chain Monte Carlo (MCMC) run for 1 million generations, including a burn‐in of 100 000, with the number of clusters (K) ranging from one to the number of nests collected (*n* = 9 in TX and 6 in FL). Each K was tested 10 times, with the most likely value of K (*i.e*., most likely number of clusters) subsequently determined using the Delta *K* method (Evanno *et al.*, 2005) implemented in the online software Structure Harvester web v0.6.94 (Earl & VonHoldt, 2012).

A total of 49 *T. difficilis* COI sequences obtained from NCBI GenBank and BOLD system were included in the mtDNA analysis (Table S2). To avoid redundancy, identical sequences collected from the same country and year were merged and represented by a single sequence in the final dataset, resulting in 27 unique representative sequences. A phylogenetic tree based on truncated sequences was constructed using MrBayes v.3.2.7 (Ronquist *et al.*, 2012), with the generalized time‐reversible model with gamma‐distributed rate variation across sites and a proportion of invariable sites utilized as the evolutionary model. Two parallel MCMC simulations were run for 2 × 10^6^ generations by using four chains (three heated and one cold), with each run sampled every 500 generations. *Technomyrmex brunneus* Forel, 1895 was included as outgroup species (Table S2; Putri & Cronin, 2023). The final tree was visualized with Interactive Tree of Life (iTOL) v6 (Letunic & Bork, 2021).

### Breeding structure analysis

Relatedness (*r*) of workers within nests were estimated using COANCESTRY v.1.0.1.9 (Wang, 2011), following algorithms described by Queller & Goodnight (1989). To account for differences in allele frequencies between regions, relatedness was calculated separately for the Texas and Florida populations.

### Behavioral trials

Workers collected from each nest were transported to the laboratory for aggression assays. Aggression assays between nests were conducted to determine the level of aggression between paired ants. Workers from two different nests were placed in a same 5 cm diameter Petri dish, with its inner sides coated with Fluon^TM^, for 5 min. Interactions between the paired ants were scored based on the scale of Suarez *et al.* (1999) with some modification: 1 = short antennation (≤ 2 s); 2 = prolonged antennation (> 2 s); 3 = aggression (lunging and attempts at biting); and 4 = fighting (using mandibles to bite or carry the other individual). In each trial, the highest level of aggression was recorded, and the average aggression score between nests was computed using the mean of the five trials conducted for each nest pairing. The test was replicated five times for each nest pair using different ants each time, as well as intra‐nest controls. A Mann–Whitney *U* test was used to compare the aggression level between Texas and Florida populations.

### Chemical analysis

Chemical analysis was used to compare the cuticular hydrocarbon (CHC) profiles of ants from different nests of the Texas and Florida populations. The level of variation within these populations was evaluated to determine if the results were consistent with the findings of the aggression assay. For each nest, a total of eight workers were frozen for 15 min at −20 °C followed by extraction in 200 *µ*L hexane containing 100 ng octacosane (n‐C28) as an internal standard. Extraction was performed for 5 min with intermittent gentle mixing. The extracts were dried under a stream of high‐purity nitrogen, reconstituted in 25 *µ*L of hexane, and transferred to a 250 *µ*L insert in a 1.5 mL auto‐injection vial. A sample volume of 2 *µ*L was injected in splitless mode utilizing a 7693B Agilent autosampler into a HP‐5MS UI column (30 m × 0.25 mm × 0.25 *µ*m; Agilent). The carrier gas used was ultrahigh‐purity helium at a constant flow rate of 0.75 mL/min. The column temperature was maintained at 50 °C for 1 min, followed by an increase to 320 °C at a rate of 10 °C/min and a final hold at 320 °C for 10 min. Chemical compounds were ionized by electron impact ionization at 70 eV and mass spectra were obtained by scanning from 40 to 550 *m*/*z* at 2.9 scans/s. The chemical profile of each individual was analyzed by determining the relative abundance of each compound. Only compounds occurring in at least 10 samples were included in the final analysis.

We used the ade4 R package (Dray & Dufour, 2007; R Core Team, 2022) to perform principal component analysis (PCA) for visualizing the chemical differentiation between colonies and populations. The level of CHC differentiation between each pair of colonies was calculated using the Euclidean distance between colony centroids. We computed the level of CHC variation within each colony by determining the mean Euclidean distance between the centroid of the nest and each of the eight workers. The between‐colony and between‐population analyses were performed on the first two PC's from the PCA. The correlation between CHC variation and genetic diversity (expected heterozygosity) within colonies, between CHC variation and genetic differentiation (*F*_ST_) between colonies, and between CHC variation and aggression level between colonies were compared using Student's *t*‐distribution for Pearson's correlation coefficient.

## Results

### Population genetic structure

Micro‐Checker analysis revealed that none of the 12 microsatellite markers in the Texas population exhibited null alleles. However, in the Florida population, four markers indicated the presence of null alleles. Upon further investigation (see the breeding structure analysis below), this was attributed to inbreeding rather than true null alleles. Therefore, all 12 markers were utilized for downstream analysis. The 12 microsatellite markers used in this study contained an average of 2.6 alleles (range: 2‒5) (Table S3). The average number of alleles present in each population was 2.08 (range: 1‒5) and 2.25 (range: 1‒3) for Texas and Florida, respectively. Additionally, we found no significant difference in allelic diversity between the two populations (Mann–Whitney *U* test, *U* = 56.00, *P* = 0.32).

**Fig. 2:**
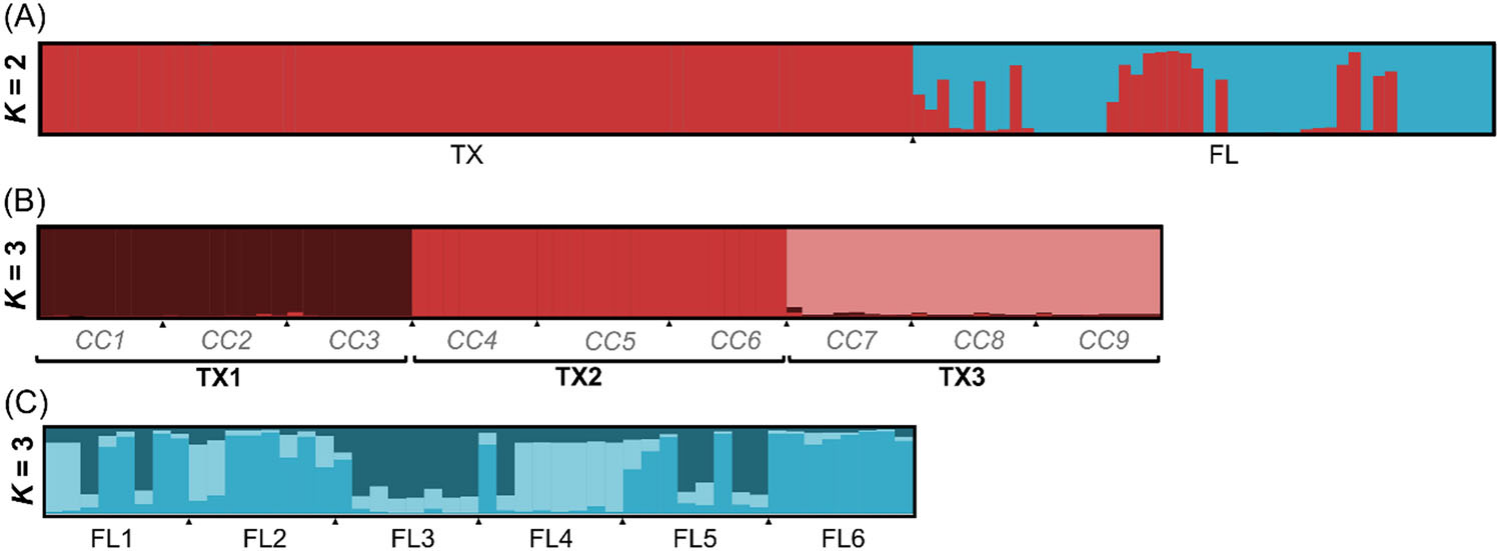
(A) Population genetic structure based on Bayesian clustering analysis of *Technomyrmex difficilis* when analyzing Texas and Florida together (*K* = 2 for all sampled workers in all nests collected). *K* = 3 was the best supported grouping for both (B) Texas and (C) Florida populations when analyzing those populations independently.

Our STRUCTURE analysis grouped samples into two genetic clusters, though these clusters did not align perfectly with the Texas and Florida populations (*K* = 2) (Fig. 2A). Within Texas, the population was further divided into three subpopulations (*K* = 3) (Fig. 2B). Group 1 (TX1) consisted of CC1, CC2, and CC3. Group 2 (TX2) contained CC4, CC5, and CC6. Group 3 (TX1) included CC7, CC8, and CC9. However, this grouping did not entirely match the results of the *G*‐test (Table S4). While some nest pairs showed no significant genetic differentiation, consistent with the results from the STRUCTURE analysis, others displayed conflicting results. Despite this, each group had private alleles, meaning certain alleles were found only in that group and not in others. This pattern indicates genetic distinctiveness among the groups and provides additional support for treating them as separate colonies. After grouping nine nests into three groups (colonies), AMOVA tests showed the variation among groups is 18% (*F*_ST_ = 0.178) (Table S5). Also, the *G‐*test among pairs of three groups showed significant genetic differentiation (Table S6). The subsequent analyses used the level of colonies (three groups) in the Texas population. In the Florida population, STRUCTURE analysis indicated that all samples were grouped into three genetic clusters, with each nest exhibiting shared genetic assignment (*K* = 3) (Fig. 2C). Also, the *G*‐test results revealed only two nest pairs FL4 and FL6 and FL3 and FL6 showed significant genetic differentiation while all other pairs did not (Table S4). However, due to the geographic distance between nests (> 1 km) it is unlikely that any pair of nests belonged to the same colony (Fig. 1). The results of AMOVA showed that 12% of the total variation (Table S7) was attributed to differences between the Texas and Florida populations (*F*_ST_ = 0.118) which indicates moderate genetic variance between the two populations. This genetic differentiation may be explained by the presence of private alleles, with three alleles found in the Texas population and seven in the Florida population (Table S8).

**Fig. 3:**
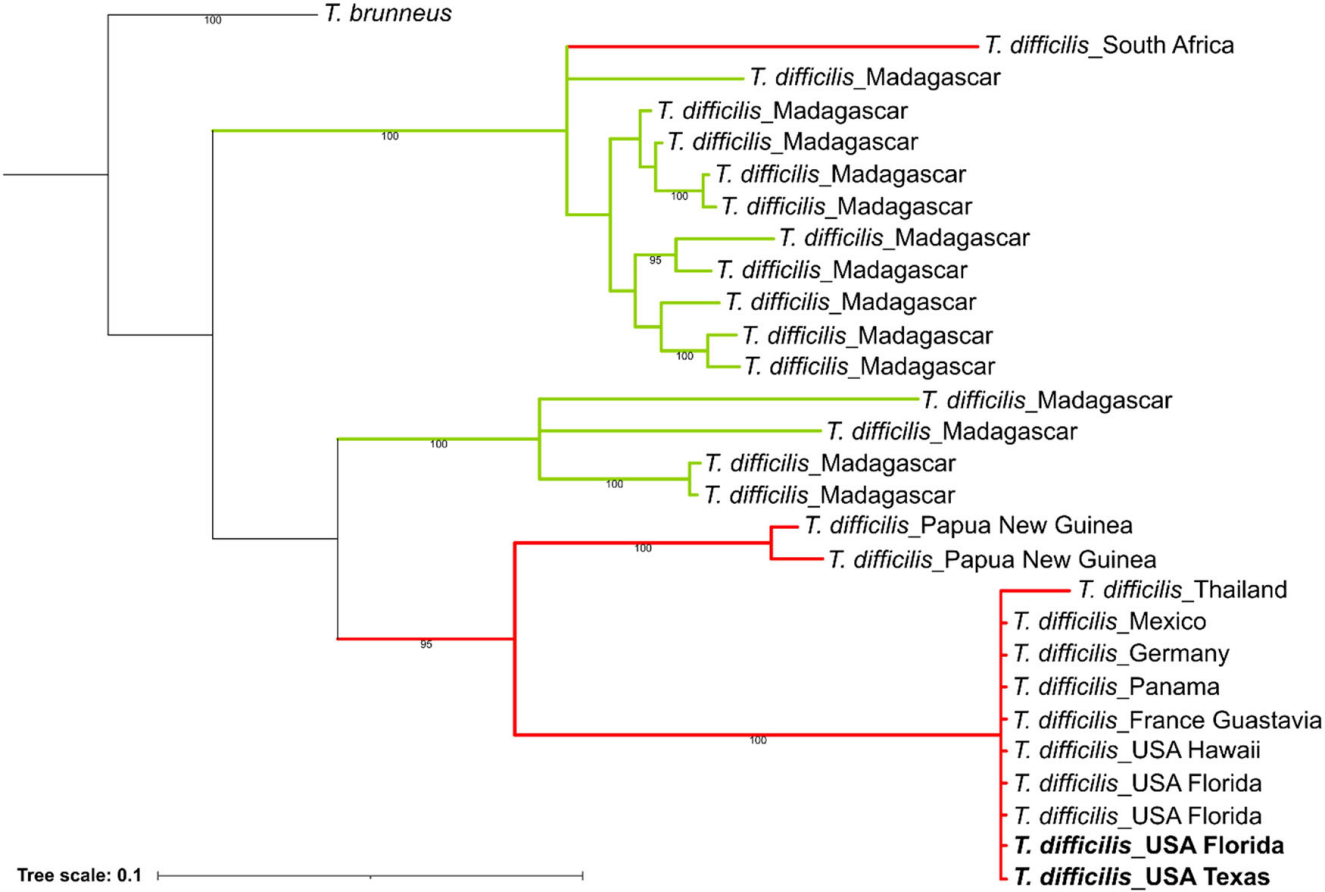
Bayesian inference tree with posterior probability below branches (only display over 90%) based on 27 COI sequences (543 bp) of *Technomyrmex difficilis* from various localities, and one outgroup species *T. brunneus* (see Table S2 for GenBank accession numbers and BOLD sample ID). The red branches indicate invasive populations, and the green branches represent native populations. Bold denotes haplotypes identified in this study.

A Bayesian phylogenetic tree was constructed using 28 cytochrome oxidase I (COI) sequences (543 bp), including 27 sequences of *Technomyrmex difficilis* and one sequence of *T. brunneus*, which was used as an outgroup (Fig. 3). The dataset included *T. difficilis* samples from invasive populations in South Africa, Papua New Guinea, Thailand, France, Mexico, Panama, Germany, Hawaii, as well as from Florida and Texas. The resulting tree showed that sequences from France, Mexico, Panama, Germany, Hawaii, Florida, and Texas were identical and clustered together, indicating that a single dominant mitochondrial haplotype has spread widely across invaded regions. Notably, sequence from South Africa grouped closer with those from Madagascar, which is considered the native range of *T. difficilis*.

### Breeding structure within each population

The coefficient of relatedness between workers within colonies did not differ significantly between the two populations (Texas, *r* = 0.274; Florida, *r* = 0.186; Mann–Whitney *U* test, *U* = 5.500, *P* = 0.364). The relatively low relatedness might indicate the presence of multiple queens within the colonies. This is further supported by worker genotypes, which are inconsistent with reproduction by a single queen, evidenced by the presence of multiple homozygous genotypes in both populations (Table S8). Additionally, values of relatedness were significantly greater than zero (TX: *t* = 4.360, *df* = 2, *P* = 0.049; FL: *t* = 4.449, *df* = 5, *P* = 0.007), as would be expected under random associations of a high number of queens or individuals moving freely among nests within the population. A negative *F*_IS_ value (*F*_IS_ = −0.088) was found in the Texas population (Table 2). Workers with low relatedness but a negative *F*_IS_ value may suggest queens mated with unrelated males. In comparison, a positive *F*_IS_ value (0.306) was found in the Florida population indicating potential inbreeding within colonies.

**Table 2:** The number of alleles (*N*_A_), allele richness (*Ar*), observed (*H*_O_), and expected (*H*_E_) heterozygosity, inbreeding coefficient (*F*_IS_) and fixation index (*F*_ST_) in the Texas (TX) and the Florida (FL) populations across 12 microsatellite loci of *Technomyrmex difficilis*

	*N*_A_	*Ar*	*H*_O_	*H*_E_	*F*_IS_	*F*_ST_
TX, *n* = 3	1.778	1.628	0.272	0.249	−0.088	0.178
FL, *n* = 6	1.736	1.576	0.151	0.204	0.261	0.128

### Comparison of aggression and CHCs

**Fig. 4:**
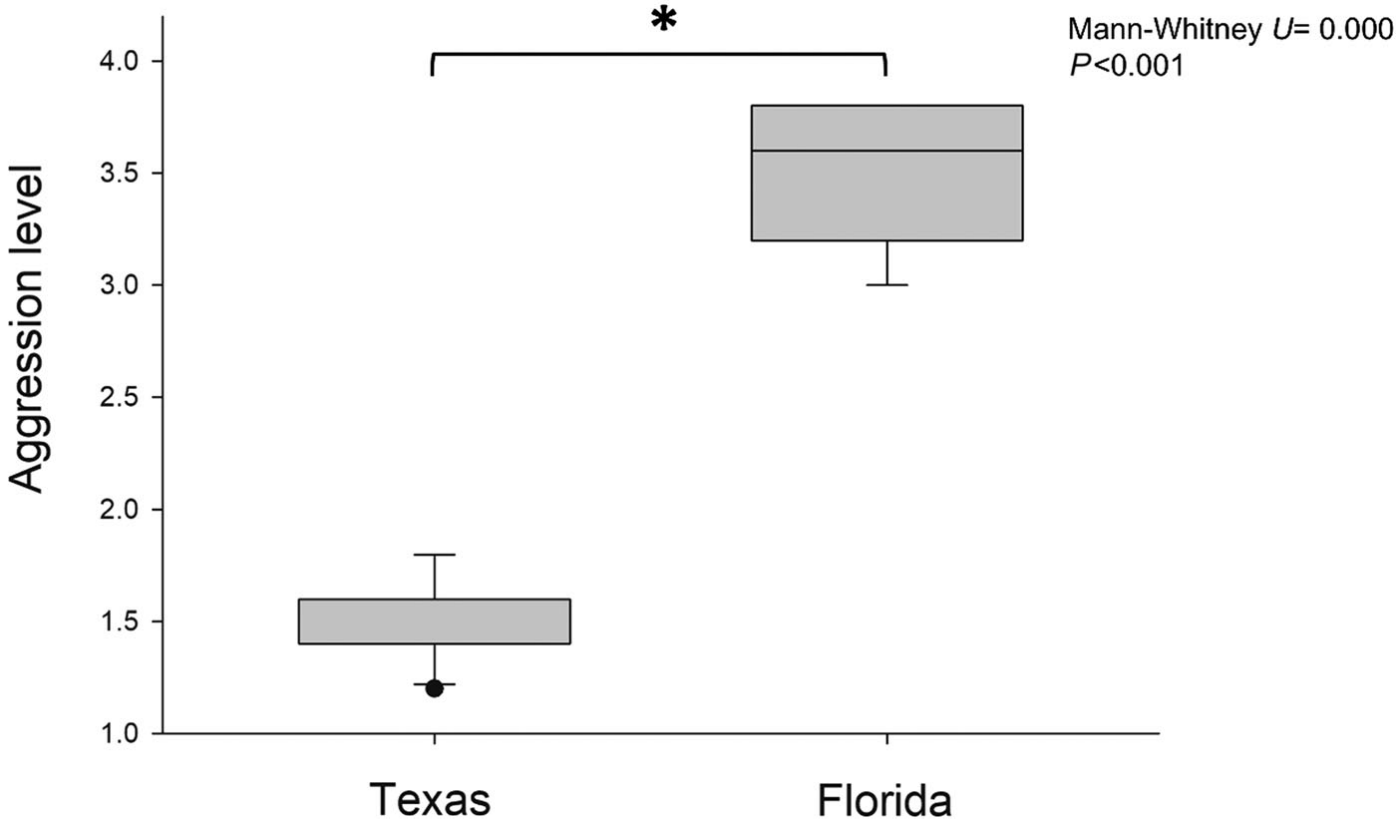
Level of aggression among non‐colony‐mate workers of *Technomyrmex difficilis* within populations. Aggression level is scaled from 1 to 4. Score of 1 and 2 referred to non‐aggressive behavior; score of 3 and 4 referred to aggressive behavior.

In the Texas population, non‐nestmates did not exhibit aggression toward each other (Chi‐square = 3.156, *P* = 0.532). Similarly, when grouped into colonies based on STRUCTURE and private alleles analysis, white‐footed ant workers did not display aggressive behaviors toward non‐colony‐mates (mean ± SE = 1.50 ± 0.04; Chi‐square = 3.002, *P* = 0.223), and the aggression within colony was also low (control: mean ± SE = 1.37 ± 0.03). In contrast, the Florida population exhibited a high level of aggression toward non‐nestmates (mean ± SE = 3.48 ± 0.33; Chi‐square = 1.575, *P* = 0.904), while within‐nest aggression remained low (mean ± SE = 1.33 ± 0.06). Due to the large geographic distances between nests and the consistently high inter‐nest aggression, each nest in the Florida population was treated as a separate colony. Colonies from the Florida population exhibited significantly stronger aggression toward non‐colony‐mate workers compared to those from the Texas population (Fig. 4, Mann–Whitney *U* test, *U* = 0.000, *P* < 0.001).

**Fig. 5:**
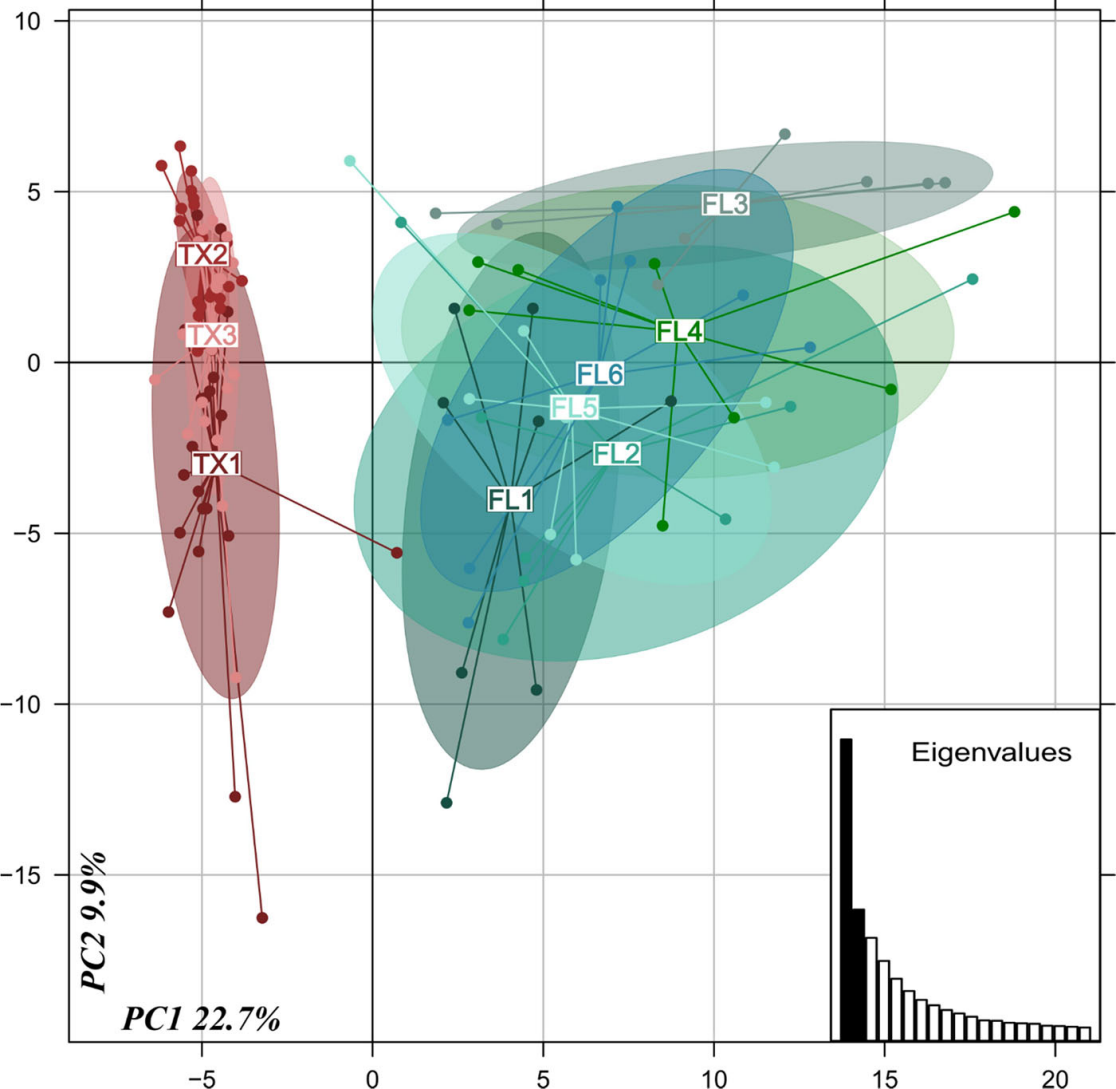
Clustering of nests in Texas and Florida using a PCA on each nest's CHC profile.

To shed some light on the observed differences in aggression levels between the Texas and Florida populations, we analyzed the chemical profiles of workers from each location. Chemical analysis revealed a clear segregation of nests between the Texas and Florida populations (Fig. 5). Intra‐population analysis revealed little variation of the chemical blend within the Texas population, while the Florida population exhibited comparatively high variation among workers. The results corroborate and provide support for the differences in aggression levels both within and between populations, as workers in the Florida populations had more variable chemical profiles and higher aggression levels. This suggests that more uniform chemical profiles among the Texas population may facilitate lower levels of aggression as workers are more likely to identify non‐nestmates as nestmates.

### Correlation between chemical variation, aggression level, and genetic variation

**Fig. 6:**
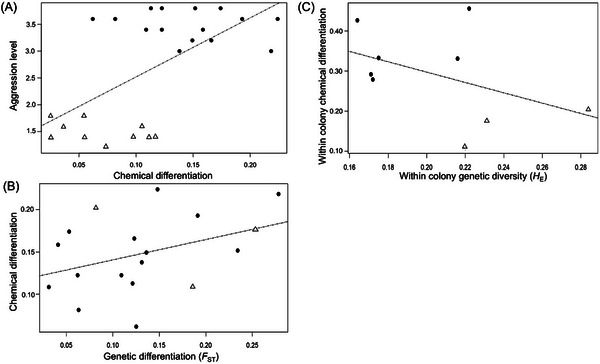
Relationships between aggression levels, chemical diversity, and genetic differentiation for pairs of colonies in the Texas (open triangle) and Florida (circle) populations. (A) Pairwise comparisons of aggression level against chemical differentiation for the overall dataset. (B) Pairwise comparisons of chemical differentiation against genetic differentiation for the overall dataset. (C) Comparison of within nest chemical variation against the genetic diversity of the nest for the overall dataset.

Chemical differentiation between nests was positively correlated with aggression level (*r* = 0.606, *P* = 0.001) (Fig. 6A), indicating that chemical cues may play a key role in mediating nestmate recognition and triggering aggressive behavior. However, no significant correlation was revealed either between chemical differentiation and genetic differentiation (*F*_ST_) (*r* = 0.386, *P* = 0.114) (Fig. 6B), or between chemical differentiation and genetic diversity (*H_E_*) (*r* = −0.446, *P* = 0.229) (Fig. 6C), suggesting that variation in cuticular hydrocarbon profiles influencing aggression is not directly linked to underlying genetic structure or diversity.

## Discussion

The present study elucidated the population structure of *T. difficilis* in Texas and Florida populations. We found no significant difference in genetic diversity between the Texas and Florida populations. Both populations exhibited low within‐colony relatedness and limited allelic variation, yet differed in their breeding strategies, aggression behaviors, and chemical profiles. Although our Florida samples were collected from sites spread across southeast Florida, the limited number of samples (*n* = 6) may not fully capture the genetic and behavioral variability of the regional population. Likewise, all nine nests from the Texas population, the only population known in Texas, were collected from a single large building. While this represents a small area, nests were sampled intensively over a small spatial scale allowing us to investigate the spatial expanse of colonies in this population. Future studies with fine‐scale and intensive sampling in Florida are needed to fully assess colony structure and reproductive strategies. As such, while the observed differences between populations are informative, caution is warranted when extrapolating these results to the species’ broader range. Despite these limitations, genetic clustering and mitochondrial data revealed consistent patterns: STRUCTURE analysis and shared mitochondrial haplotypes suggest that the Texas population may have originated from Florida, consistent with its known history. Interestingly, while Florida colonies showed evidence of inbreeding and high inter‐colony aggression, Texas colonies exhibited potential localized outbreeding and low aggression, possibly facilitated by more uniform cuticular hydrocarbon profiles.

Genetic bottlenecks happen when a new population is founded by a small number of individuals, leading to a loss of genetic diversity through genetic drift. This effect is stronger if all colonists come from the same source population and can be intensified by slow population growth or prolonged small population sizes (Nei *et al.*, 1975; Chakraborty & Nei, 1977). Thus, a newly established population is likely to have significantly lower genetic diversity than its source population. However, our results showed that the genetic diversity of Florida population was not significantly higher than Texas population. We had expected higher genetic diversity in Florida, assuming it was colonized earlier and is believed to have been the source of the Texas population. A similar pattern has been observed in the red‐imported fire ant (*Solenopsis invicta* Buren, 1972), which first invaded the southern United States from South America and later spread to Taiwan through secondary introduction (Ascunce *et al.*, 2011). Interestingly, comparisons of genetic diversity between Southern US and Taiwanese population revealed similar levels of genetic diversity as measured by *N_A_* (number of alleles) and *H_E_* (expected heterozygosity) (Yang *et al.*, 2008). Likewise, the big‐headed ant (*Pheidole megacephala* (Fabricius, 1793)) also showed comparable genetic diversity (*Ar*; allele richness) between its introduced populations in Australia and Taiwan region (Liu *et al.*, 2022). Given that the Texas population was likely introduced from Florida, the similar levels of genetic diversity between the two populations suggest that this introduction event involved sufficient genetic variation to avoid a strong bottleneck effect.

Our results showed that the Texas and Florida populations share a single mtDNA haplotype. Phylogenetic reconstruction further revealed that this haplotype is also present in two additional *T. difficilis* sequences from unsampled regions of Florida, supporting a Florida origin for the Texas population but not necessarily from the specific localities we sampled. Notably, this same haplotype has also been identified in other introduced populations, including those in Gustavia (France), Veracruz (Mexico), Barro Colorado Island (Panama), Brandenburg (Germany) and, Hawaii (Fig. 3), suggesting that this lineage is widespread across invaded regions. Although we detected some alleles in Texas that were absent from our sampled Florida nests, these are best interpreted as reflecting unsampled genetic diversity within Florida rather than an independent origin. Such patterns are commonly observed in invasive species, where founder effects and repeated introductions contribute to the widespread distribution of a single dominant haplotype (Vogel *et al.*, 2010; Ascunce *et al.*, 2011). In contrast, the *T. difficilis* sample from Papua New Guinea, Thailand, and South Africa formed distinct lineages respectively, suggesting a different maternal origin or an independent introduction event to those countries. These sequences represent all currently available *T. difficilis* COI records in the NCBI and BOLD database, underscoring the limited geographic coverage of existing genetic data. Future studies incorporating additional samples, particularly from the native range and other invaded regions, will be essential to determine whether the dominant haplotype reflects a truly widespread maternal lineage or is the result of sampling gaps.

Report from a staff member at the Texas State Aquarium indicated that the initial *T. difficilis* infestation coincided with the arrival of a potted palm tree imported from Florida. Consistent with this observation, we also found that most nests in the Texas population were concentrated in potted palm trees, often with dense worker activity. Our genetic analyses also support a Florida origin. The STRUCTURE analysis showed that workers from the Texas population cluster genetically with some individuals from the Florida population, and they shared the same mitochondrial haplotype as described previously. While our Florida sampling was geographically limited, the combination of genetic clustering and circumstantial evidence suggests that the Texas population most likely originated from Florida. Altogether, these findings highlight the role of human‐mediated transport (the movement of ornamental plants in our case) in facilitating the spread of *T. difficilis*.

Both the Texas and Florida populations showed low within‐colony relatedness, which is often associated with the presence of multiple reproductive queens. However, the two populations appear to follow different breeding strategies. The colony founding strategy of *T. difficilis* is unique. Colonies initially founded by winged females after nuptial flights, but subsequent reproduction is taken over by inbred, wingless intercastes that mate with related males within the nest (Yamauchi *et al.*, 1991). The negative inbreeding coefficient observed in the Texas population suggests queens are mating with unrelated males, which could indicate that this population is still in an early stage of colony development where reproduction is dominated by outcrossing alate queens rather than inbred intercastes. Such outbreeding could occur if founding queens originated from multiple genetically distinct source nests or if they mated with unrelated males from neighboring colonies before territorial boundaries were fully established. This type of outbreeding can provide important fitness benefits, as it reduces the likelihood of queens mating with males that carry the same allele at the sex‐determining locus, thereby avoiding the production of diploid males which is a costly outcome for colony fitness. In fire ants (*Solenopsis invicta*), diploid male production has been shown to increase colony mortality and hinder growth, particularly in monogyne colonies where such males are produced early in development (Ross & Fletcher, 1986). Similar negative effects of diploid male production, including reduced fitness and lower survival, have also been reported in bumblebees (Whitehorn *et al.*, 2009). This localized outbreeding may help maintain genetic diversity despite the presence of budding in this species.

In contrast, the Florida population shows a positive inbreeding coefficient, suggesting that queens may mate with related males. Although worker relatedness is relatively low, this is likely due to reproduction by multiple reproductives within colonies. Warner (2003) reported that the presence of intercastes in white‐footed ant colonies in Florida and dispersal through budding, a strategy often associated with inbreeding due to limited dispersal and local mating. Similar breeding patterns have been observed in related species. *Technomyemex brunneus* and *Technomyrmex vitiensis* Mann, 1921 have comparable strategies, in which intercastes mate with ergatoid males and expand the colony through budding (Yamauchi *et al.*, 1991; Oettler & Heinze, 2009). Similarly, in the Asian needle ant (*Brachyponera chinensis* (Emery, 1895)), colonies are initiated by a single queen but later but later produce daughter queens that reintegrate into their natal nest and preferentially mate with related males (Eyer *et al.*, 2018). The worker relatedness observed in *B. chinensis* (*r* = 0.202) closely matches that of the Florida *T. difficilis* population (*r* = 0.186).

In such systems, inbreeding may promote the development of large, polydomous colonies by maintaining a stable kin structure, where genetic similarity among nestmates supports social cohesion and cooperative behavior, even in the presence of multiple reproductive queens (Haag‐liautard *et al.*, 2009; Helanterä *et al.*, 2009). Furthermore, in environments where opportunities for dispersal or outbreeding are limited (e.g., due to isolation or competition), inbreeding may be favored as the only viable reproductive strategy, the short‐term benefit of ensuring reproductive continuity may outweigh the genetic costs associated with inbreeding, especially in species where budding and local expansion are more advantageous than risky dispersal (Bourke & Heinze, 1994; Heinze & Tsuji, 1995). In our case, we did not collect any queens, intercastes, or males during this study, which may reflect seasonal, geographic, or sampling limitations. Further studies involving field observations of reproductive castes are needed to better understand the breeding system of *T. difficilis* and its potential role in the invasion success of this species.

The Florida population exhibited high levels of inter‐colony aggression, which may be influenced by genetic structure or localized inbreeding within colonies, though the specific causes remain uncertain. In contrast, colonies from the Texas population showed lower levels of aggression between each other, potentially reducing barriers to reproductive and worker interactions among neighboring colonies. This behavioral tolerance could increase the chances of queens encountering unrelated males during mating thereby facilitating localized outbreeding. However, given that the two populations were sampled over different spatial scales, it is difficult to determine whether these behavioral differences are driven by genetic, environmental, or variation in nest spacing and local habitat configuration. Despite these behavioral differences, both populations exhibited low genetic diversity. To better understand whether chemical communication may be involved, we examined the relationship between genetic and chemical variation. It is important to note that the genetic diversity discussed here refers to genome‐wide neutral variation, specifically microsatellite markers, whereas the synthesis of cuticular hydrocarbons is enzyme‐regulated, with these enzymes being controlled by specific genes (Blight *et al.*, 2012). Interestingly, the observed aggression patterns in Texas and Florida could not be explained by correlations between genetic differentiation and chemical variation. No significant association was found between genetic differentiation (*F*_ST_) and chemical distance, either within or between the Texas and Florida populations. Furthermore, there was no clear relationship between genetic diversity (*H*_E_) and chemical distance within these populations (Fig. 6). While neutral genetic markers did not reveal significant differences among colonies, loci associated with odor and behavioral traits may vary among colonies. Similar findings have been reported in other invasive ants. For example, Schmidt *et al.* (2010) observed low intraspecific aggression and consistent cuticular hydrocarbon profiles despite high genetic differentiation in pharaoh ants (*Monomorium pharaonis* (Linnaeus, 1758)). Conversely, Blight *et al.* (2012) reported weak genetic differentiation between two supercolonies of the Argentine ant, yet their chemical profiles were qualitatively distinct. These findings suggest that genetic diversity does not always align with chemical profile diversity in invasive ant populations. Variation in chemical profiles can arise from environmental factors, genetic factors, or their interplay. For instance, diet has been shown to affect nestmate recognition in Argentine ants; workers from the same colony but raised on different diets exhibit aggression toward one another (Liang & Silverman, 2000). Similarly, Sorvari *et al.* (2008) found that aggression in *Formica aquilonia* increased when both the proportion of dietary ingredients and overall food availability were altered. In the case of the Texas and Florida populations of *T. difficilis* studied here, environmental factors may influence aggression behaviors. Texas ants, all collected from the grounds of the Texas State Aquarium, exhibited low aggression levels, while Florida ants, collected from more spatially dispersed and ecologically varied locations, showed higher aggression levels. Because the populations were sampled under different conditions, we cannot definitively attribute these behavioral patterns to environmental influences alone. This underscores the need to further investigate how environmental context may shape chemical profiles and aggression dynamics.

In conclusion, we present the first integrated study of behavior, chemical recognition, and population genetics on *T. difficilis*. Our findings reveal important differences between the Texas and Florida populations, including breeding system differences, behavioral variation, and chemical profile distinctions. The Texas population exhibited lower inter‐colony aggression, more uniform chemical profiles, and a negative inbreeding coefficient, suggesting the possibility of localized outbreeding. In contrast, the Florida population showed signs of inbreeding, higher inter‐colony aggression, and greater chemical diversity, consistent with stronger territoriality. Despite behavioral divergence, there was no significant association between genetic differentiation and chemical profile variation, suggesting that aggression patterns may be shaped by environmental factors or genetic makeup at specific loci related to cuticular hydrocarbons. These findings provide a foundation for future work on population structure, reproductive strategy, and invasion pathways of *T. difficilis*, and highlight the need for broader geographic sampling and deeper taxonomic resolution within the genus *Technomyrmex*.

## Disclosure

The authors declare that they have no conflict of interest.

## Supporting information




**Table S1** PCR primers tested for *Technomyrmex difficilis*.
**Table S2** Sample information, GenBank accession numbers, and BOLD sample ID for COI sequences used to construct the phylogenetic tree in this study.
**Table S3** The number of alleles (*N*_A_), allele richness (*Ar*), observed (*H_O_*) and expected (*H_E_*) heterozygosity, inbreeding coefficient (*F*_IS_) and fixation index (*F*_ST_) for each of the 12 microsatellite loci in Texas (TX), Florida (FL) populations, and all samples in this study.
**Table S4** *G‐*test results for nest pairs in Texas (TX) and Florida (FL) populations with Bonferroni‐corrected significance levels (*P* < 0.0014 for Texas, *P* < 0.003 for Florida).
**Table S5** Hierarchical partitioning of genetic diversity using an analysis of molecular variance (AMOVA) for Texas population.
**Table S6** *G‐*test results for colony pairs in Texas (TX) population after grouping nine nests into three colonies with Bonferroni‐corrected significance levels (*P* < 0.017).
**Table S7** Hierarchical partitioning of genetic diversity using an analysis of molecular variance (AMOVA) for the overall dataset.
**Table S8** Genotypes of *Technomyrmex difficilis* workers in each colony for each of the 12 microsatellite loci.

## Data Availability

All raw sequence files have been deposited in the National Center for Biotechnology Information (NCBI) under GenBank accession numbers PV662446‒PV662485.
