# Supplementary material for: Colony and population genetic structure of the newly invasive white‐footed ant (Technomyrmex difficilis) in the United States
Source: Insect Sci. 2025 Nov 12;32(6):1954–68. doi: 10.1111/1744-7917.70196 (PMC12717334; doi:10.1111/1744-7917.70196)
Supplement: Supplementary file 1 — Table S1 PCR primers tested for Technomyrmex difficilis. Table S2 Sample information, GenBank accession numbers, and BOLD sample ID for COI sequences used to construct the phylogenetic tree in this study. Table S3 The number of alleles (N A), allele richness (Ar), observed (HO ) and expected (HE ) heterozygosity, inbreeding coefficient (F IS) and fixation index (F ST) for each of the 12 microsatellite loci in Texas (TX), Florida (FL) populations, and all samples in this study. Table S4 G‐test results for nest pairs in Texas (TX) and Florida (FL) populations with Bonferroni‐corrected significance levels (P < 0.0014 for Texas, P < 0.003 for Florida). Table S5 Hierarchical partitioning of genetic diversity using an analysis of molecular variance (AMOVA) for Texas population. Table S6 G‐test results for colony pairs in Texas (TX) population after grouping nine nests into three colonies with Bonferroni‐corrected significance levels (P < 0.017). Table S7 Hierarchical partitioning of genetic diversity using an analysis of molecular variance (AMOVA) for the overall dataset. Table S8 Genotypes of Technomyrmex difficilis workers in each colony for each of the 12 microsatellite loci. [file INS-32-1954-s001.docx]

**Supporting information**

Table S1

| Primer | Sequence (5' -3') | *Species* | Author and Year | Inclde or exclude | Excluding reason |
| --- | --- | --- | --- | --- | --- |
| L911F | ACGCCTCGTCAAGAGTGGTCTC | *Tapinoma melanocephalum* | Zheng et al. 2018 | Include |  |
| L911R | GGAAAGCAGCAATTTTCTCG |  |  |  |  |
| LP24F | GGAACAGGTGCTGAGAATCC | Leptomyrmex pallens | Berman et al. 2014 | Include |  |
| LP24R | TGGCTAGTCCATGATTGTGC |  |  |  |  |
| Ant859F | ACTACGCGGAGAAACGTCTGGT | *general ant* | Butler et al. 2014 | Include |  |
| Ant859R | GTGATCTAAACTTCGATGAAC |  |  |  |  |
| Ant7680F | ACTCCCGGAGCAGCAATTATCC | *general ant* | Butler et al. 2014 | Include |  |
| Ant7680R | TAGGACAAAATGGAGCCCGC |  |  |  |  |
| Ant5035F | ACAGGATAGTTTCGCGGTTTATGG | *general ant* | Butler et al. 2014 | Include |  |
| Ant5035R | ACTGACTCGYAGTGTATTTGAGGT |  |  |  |  |
| Ant7249F | ACAAGTGTCAAGGGCGACTGAG | *general ant* | Butler et al. 2014 | Include |  |
| Ant7249R | CGGGGACAATGGAGCAATCA |  |  |  |  |
| Ant1343F | ACTCGGTCCCGTGCCTTCGATT | *general ant* | Butler et al. 2014 | Include |  |
| Ant1343R | GRGGGCGCGTCAAATTTGCT |  |  |  |  |
| Ant8424F | ACTCATAATGCAGATGATGGAACTCCT | *general ant* | Butler et al. 2014 | Include |  |
| Ant8424R | GGCGAGTAACACAATGGCAC |  |  |  |  |
| Ant3653F | ACAGCAGAGACCAATCAACGGA | *general ant* | Butler et al. 2014 | Include |  |
| Ant3653R | GGCAATTATCGGACCGGGTT |  |  |  |  |
| Ant20F | AGGTCCTAGCAGGTAACATTG | *general ant* | Butler et al. 2014 | Include |  |
| Ant20R | CCTCGGTCGATCGAGCGAGC |  |  |  |  |
| Ant575F | TCAGGTTCGACACATGTGCC | *general ant* | Butler et al. 2014 | Include |  |
| Ant575R | TCAAGATCGTTTGTCAGGCTGA |  |  |  |  |
| Ant12220F | AAAAGAGGCGGGCGTTCTTA | *general ant* | Butler et al. 2014 | Include |  |
| Ant12220R | GGTGTTCYGCCCCACCCGTA |  |  |  |  |
| Lhum19F | ACCTCTTAAAGCAATTGCATGTGG | *Linepithema humile* | Krieger et al. 2003 | Exclude | monomorphic |
| Lhum19R | ACGATCGCGTCCTTTGAG |  |  |  | amplification |
| L1178F | ACCACAGTACCCTGGAGGCATT | *Tapinoma melanocephalum* | Zheng et al. 2018 | Exclude | non- amplification |
| L1178R | CGTGAGAGAAATTTGCGTGA |  |  |  |  |
| L1315F | ACGCATGTGTGCAGTCTCGAAT | *Tapinoma melanocephalum* | Zheng et al. 2018 | Exclude | non- amplification |
| L1315R | GGGTCTGATGGAATACCGTG |  |  |  |  |
| L434F | ACAGCTCGGCTGATTCGTTATG | *Tapinoma melanocephalum* | Zheng et al. 2018 | Exclude | non- amplification |
| L434R | TTCTTTTCACTCGTGTTGCG |  |  |  |  |
| Ant10878F | ACCGGGTGYTAGTCGTCGCCAT | *general ant* | Butler et al. 2014 | Exclude | monomorphic |
| Ant10878R | GATCAATGCCGCAACGCTAA |  |  |  | amplification |
| Ant9218F | ACGACCCACTTTGCCCTCGTAA | *general ant* | Butler et al. 2014 | Exclude | monomorphic |
| Ant9218R | CTCTCGATTAGTCAGGGTGGC |  |  |  | amplification |
| Ant3648F | ACCTCCTGGTCCTGGATCTCCA | *general ant* | Butler et al. 2014 | Exclude | monomorphic |
| Ant3648R | TAACACCATGCCCTCTGTCG |  |  |  | amplification |
| Ant3993F | ACTGATCCGCTCTTAAAATTTAGATGGA | *general ant* | Butler et al. 2014 | Exclude | non- amplification |
| Ant3993R | ACTTTCCGCRGCATTAAACATTTTCTT |  |  |  |  |
| Ant1368F | ACTACCCCAATGACGACACG | *general ant* | Butler et al. 2014 | Exclude | non- amplification |
| Ant1368R | CTATGCAGGTGCGGGTGTAT |  |  |  |  |
| Ant2341F | RAACAGCAGCTGTCCGGAGG | *general ant* | Butler et al. 2014 | Exclude | monomorphic |
| Ant2341R | GTCGCTGATCGCCACGTTCC |  |  |  | amplification |
| Ant2936F | GGGGGATCCGGTAATCCTCT | *general ant* | Butler et al. 2014 | Exclude | non- amplification |
| Ant2936R | TCGCCCTGCAGTTAATGTGT |  |  |  |  |
| Ant8498F | GATGCGAAGAGAGGCACGCG | *general ant* | Butler et al. 2014 | Exclude | non- amplification |
| Ant8498R | TGTTGCGAACYTAGGTGGCCTC |  |  |  |  |
| Ant9181F | TGCCACTTACGCTGTGCACAC | *general ant* | Butler et al. 2014 | Exclude | monomorphic |
| Ant9181R | AAATGCGGCCGAAGAGAAGA |  |  |  | amplification |
| Ant11315F | AGCGTGTGCGACCGTGTAGC | *general ant* | Butler et al. 2014 | Exclude | non- amplification |
| Ant11315R | GCCATATATCATGGCTTGCCAG |  |  |  |  |
| Ant11400F | CAACCACTTTGGGGCGCGAG | *general ant* | Butler et al. 2014 | Exclude | non- amplification |
| Ant11400R | CGAACCTCTTAATGAAATTCTCACCC |  |  |  |  |
| Ant11893F | CAGGCTCGGRACGTTAATGC | *general ant* | Butler et al. 2014 | Exclude | monomorphic |
| Ant11893R | GGTGCCGACGTCTAGCTAGC |  |  |  | amplification |
| Ant21F | TTCTCGGGAGCAACCGTGGT | *general ant* | Butler et al. 2014 | Exclude | monomorphic |
| Ant21R | CCATCACGCACTCCACCTCG |  |  |  | amplification |
| Ant608F | AGCGGATCTAGTGGTCTTGG | *general ant* | Butler et al. 2014 | Exclude | monomorphic |
| Ant608R | ATGGAGGGGAGTAAGAGCGA |  |  |  | amplification |
| Ant1387F | ATAGGTGCCACATACGCGTG | *general ant* | Butler et al. 2014 | Exclude | non- amplification |
| Ant1387R | CACAGCCGACTCCCCTCTCC |  |  |  |  |

Table S2 Sample information, Genbank accession numbers, and BOLD sample ID for COI sequences used to construct the phylogenetic tree in this study

| Species | Location | Genbank accession no. | BOLD sample ID | Year collected |
| --- | --- | --- | --- | --- |
| *Technomyrmex difficilis* | Gustavia, France | KF765477 |  | 2011 |
| *Technomyrmex difficilis* | Brandenburg, Germany |  | DEI-GISHym5393,  DEI-GISHym5409 | 2024 |
| *Technomyrmex difficilis* | Antananarivo, Madagascar |  | CASENT0141249-D01, CASENT0141255-D01, CASENT0141258-D01 | 2008 |
| *Technomyrmex difficilis* | Antsiranana, Madagascar | DQ176116 |  | 2005 |
| *Technomyrmex difficilis* | Antsiranana, Madagascar |  | CASENT0261045-D01, CASENT0261066-D01, CASENT0261111-D01, CASENT0261155-D01, CASENT0261301-D01 | 2011 |
| *Technomyrmex difficilis* | Mahajanga, Madagascar |  | CASENT0107125-D01 | 2001 |
| *Technomyrmex difficilis* | Mahajanga, Madagascar |  | CASENT0430183-D01 | 2004 |
| *Technomyrmex difficilis* | Mahajanga, Madagascar | JN287708, JN287679, JN287684, JN287687, JN287688 |  | 2009 |
| *Technomyrmex difficilis* | Toamasina, Madagascar |  | CASENT0063910-D01, CASENT0065059-D01, CASENT0067361-D01, CASENT0067491-D01, CASENT0067513-D01,  CASENT0067535-D01,  CASENT0067542-D01,  CASENT0067675-D01,  CASENT0068237-D01, | 2005 |
| *Technomyrmex difficilis* | Veracruz, Mexico |  | BIOUG45004-G12, BIOUG44906-E01, BIOUG44906-H09, BIOUG44909-F09, BIOUG44974-E12, BIOUG44974-F06 | 2017 |
| *Technomyrmex difficilis* | Barro Colorado Is, Panama | MK758942, MK769879 |  | 2011 |
| *Technomyrmex difficilis* | Barro Colorado Is, Panama |  | YB-BCI42896 | 2019 |
| *Technomyrmex difficilis* | Papua New Guinea |  | BIOUG30790-D12 | 2015 |
| *Technomyrmex difficilis* | Papua New Guinea | PP069711 |  | 2023 |
| *Technomyrmex difficilis* | KwaZulu-Natal, South Africa |  | ETKSPS0011, ETKSPS0012, ETKSPS0013, ETKSPS0014 | 2011 |
| *Technomyrmex difficilis* | Nakhon Ratchasima, Thailand |  | BIOUG94550-D07 | 2022 |
| *Technomyrmex difficilis* | Florida, USA |  | 260806-11 | 2006 |
| *Technomyrmex difficilis* | Florida, USA |  | 10BBUFO-0140 | 2010 |
| *Technomyrmex difficilis* | Florida, USA | PV662469-PV662485 |  | 2022 |
| *Technomyrmex difficilis* | Hawaii, USA | HQ925251, HQ925252 |  | 2008 |
| *Technomyrmex difficilis* | Texas, USA | PV662446-PV662468 |  | 2021 |
| *Technomyrmex brunneus* | Ogasawara, Japan | OP435370 |  | 2022 |

Table S3 The number of alleles (*N*_A_), allele richness (*Ar*), observed (*H_O_*) and expected (*H_E_*) heterozygosity, inbreeding coefficient (*F*_IS_) and fixation index (*F*_ST_) for each of the 12 microsatellite loci in Texas (TX), Florida (FL) populations, and all samples in this study.

| Marker | ***N*_A_** | ***Ar*** | ***H*_O_** | ***H*_E_** | ***F*_IS_** | ***F*_ST_** |
| --- | --- | --- | --- | --- | --- | --- |
| *TX* |  |  |  |  |  |  |
| Ant859 | 1 | 1.000 | 0.000 | 0.000 | 0.000 | 0.000 |
| Ant8424 | 2 | 2.000 | 0.437 | 0.451 | 0.041 | 0.009 |
| Ant12200 | 1 | 1.000 | 0.000 | 0.000 | 0.000 | 0.000 |
| LP24 | 2 | 2.000 | 0.450 | 0.432 | -0.047 | -0.003 |
| Ant3659 | 5 | 5.000 | 0.678 | 0.682 | 0.105 | 0.100 |
| Ant1343 | 3 | 2.574 | 0.017 | 0.033 | 0.498 | 0.925 |
| Ant7680 | 2 | 2.000 | 0.159 | 0.135 | -0.181 | 0.085 |
| Ant20 | 2 | 2.000 | 0.325 | 0.249 | -0.309 | 0.091 |
| Ant575 | 2 | 2.000 | 0.236 | 0.198 | -0.195 | 0.057 |
| Ant5035 | 1 | 1.000 | 0.000 | 0.000 | 0.000 | 0.000 |
| L911 | 2 | 2.000 | 0.357 | 0.409 | 0.128 | -0.009 |
| Ant7424 | 2 | 2.000 | 0.597 | 0.409 | -0.459 | 0.051 |
| *FL* |  |  |  |  |  |  |
| Ant859 | 2 | 1.984 | 0.042 | 0.042 | -0.011 | -0.015 |
| Ant8424 | 3 | 3.000 | 0.313 | 0.411 | 0.287 | 0.101 |
| Ant12200 | 3 | 2.978 | 0.107 | 0.131 | 0.384 | 0.074 |
| LP24 | 2 | 2.000 | 0.155 | 0.275 | 0.505 | 0.109 |
| Ant3659 | 3 | 3.000 | 0.551 | 0.592 | 0.195 | 0.088 |
| Ant1343 | 2 | 2.000 | 0.000 | 0.113 | 1.000 | 0.054 |
| Ant7680 | 1 | 1.000 | 0.000 | 0.000 | 0.000 | 0.000 |
| Ant20 | 1 | 1.000 | 0.000 | 0.000 | 0.000 | 0.000 |
| Ant575 | 3 | 2.975 | 0.111 | 0.108 | -0.032 | -0.003 |
| Ant5035 | 2 | 2.000 | 0.201 | 0.175 | 0.237 | 0.189 |
| L911 | 3 | 3.000 | 0.103 | 0.396 | 0.750 | 0.271 |
| Ant7424 | 2 | 2.000 | 0.212 | 0.196 | -0.118 | -0.021 |
| *All* |  |  |  |  |  |  |
| Ant859 | 2 | 1.572 | 0.018 | 0.018 | -0.014 | 0.019 |
| Ant8424 | 3 | 2.911 | 0.395 | 0.459 | 0.141 | 0.005 |
| Ant12200 | 3 | 2.421 | 0.025 | 0.042 | 0.381 | 0.041 |
| LP24 | 2 | 2.000 | 0.331 | 0.383 | 0.128 | 0.033 |
| Ant3659 | 5 | 4.998 | 0.632 | 0.741 | 0.138 | 0.033 |
| Ant1343 | 3 | 2.336 | 0.009 | 0.345 | 0.975 | 0.053 |
| Ant7680 | 2 | 1.991 | 0.096 | 0.091 | -0.077 | 0.058 |
| Ant20 | 2 | 2.000 | 0.195 | 0.176 | -0.185 | 0.132 |
| Ant575 | 3 | 2.705 | 0.188 | 0.172 | -0.105 | 0.035 |
| Ant5035 | 2 | 1.990 | 0.043 | 0.058 | 0.235 | 0.085 |
| L911 | 3 | 3.000 | 0.274 | 0.590 | 0.432 | 0.325 |
| Ant7424 | 2 | 2.000 | 0.468 | 0.368 | -0.329 | 0.094 |

Table S4 *G-*test results for nest pairs in Texas (TX) and Florida (FL) populations with Bonferroni-corrected significance levels (p < 0.0014 for Texas, p < 0.003 for Florida).

| Nests pair | Chi^2^ | d.f. | P- value |
| --- | --- | --- | --- |
| CC5 & CC6 | 5.989 | 12 | 0.917 |
| CC7 & CC8 | 12.128 | 16 | 0.735 |
| CC7 & CC9 | 15.147 | 16 | 0.514 |
| CC1 & CC2 | 17.077 | 16 | 0.381 |
| CC2 & CC7 | 19.155 | 16 | 0.261 |
| CC4 & CC6 | 15.239 | 12 | 0.229 |
| CC8 & CC9 | 15.416 | 12 | 0.219 |
| CC2 & CC3 | 20.581 | 16 | 0.195 |
| CC4 & CC5 | 16.900 | 12 | 0.153 |
| CC2 & CC8 | 23.003 | 16 | 0.114 |
| CC1 & CC3 | 23.346 | 16 | 0.105 |
| CC2 & CC5 | 26.194 | 16 | 0.051 |
| CC2 & CC9 | 26.716 | 16 | 0.045 |
| CC2 & CC6 | 27.812 | 14 | 0.015 |
| CC1 & CC7 | 34.035 | 18 | 0.012 |
| CC1 & CC4 | 33.021 | 16 | 0.007 |
| CC3 & CC4 | 35.534 | 16 | 0.003 |
| CC1 & CC8 | 38.843 | 18 | 0.003 |
| CC5 & CC9 | 35.975 | 16 | 0.003 |
| CC2 & CC4 | 37.530 | 16 | 0.002 |
| CC3 & CC7 | 41.479 | 18 | 0.001 |
| CC3 & CC9 | 42.489 | 18 | <0.001 |
| CC1 & CC9 | 43.554 | 18 | <0.001 |
| CC3 & CC5 | 41.120 | 16 | <0.001 |
| CC6 & CC9 | 39.271 | 14 | <0.001 |
| CC3 & CC6 | 42.986 | 16 | <0.001 |
| CC4 & CC9 | 46.856 | 16 | <0.001 |
| CC1 & CC6 | 47.909 | 16 | <0.001 |
| CC5 & CC8 | 48.298 | 16 | <0.001 |
| CC3 & CC8 | 52.119 | 18 | <0.001 |
| CC6 & CC8 | 46.243 | 14 | <0.001 |
| CC1 & CC5 | 50.669 | 16 | <0.001 |
| CC6 & CC7 | 53.042 | 16 | <0.001 |
| CC5 & CC7 | 57.595 | 18 | <0.001 |
| CC5 & CC7 | 60.967 | 18 | <0.001 |
| CC4 & CC8 | 61.528 | 16 | <0.001 |
| FL1 & FL2 | 10.048 | 20 | 0.967 |
| FL1 & FL5 | 11.695 | 18 | 0.863 |
| FL2 & FL2 | 13.288 | 18 | 0.774 |
| FL5 & FL6 | 12.129 | 16 | 0.735 |
| FL1 & FL4 | 16.076 | 18 | 0.587 |
| FL2 & FL4 | 19.524 | 18 | 0.360 |
| FL2 & FL6 | 18.151 | 16 | 0.315 |
| FL1 & FL3 | 23.058 | 20 | 0.286 |
| FL3 & FL5 | 25.193 | 18 | 0.120 |
| FL1 & FL6 | 27.795 | 20 | 0.114 |
| FL2 & FL3 | 27.177 | 18 | 0.076 |
| FL3 & FL4 | 25.722 | 16 | 0.058 |
| FL4 & FL5 | 30.733 | 16 | 0.014 |
| FL4 & FL6 | 37.328 | 16 | 0.002 |
| FL3 & FL6 | 40.559 | 14 | <0.001 |

Table S5 Hierarchical partitioning of genetic diversity using an analysis of molecular variance (AMOVA) for Texas population.

|  | Sum of Squares | Variance component | Percentage | F-stats |
| --- | --- | --- | --- | --- |
| Among colonies | 39.042 | 0.369 | 18% | 0.178* |
| Among individuals | 124.396 | 0.103 | 5% | 0.006* |
| Within individuals | 115.000 | 1.597 | 77% | 0.228* |
| Total | 278.438 | 2.069 | 100% |  |

Table S6 *G-*test results for colony pairs in Texas (TX) population after grouping nine nests into three colonies with Bonferroni-corrected significance levels (p < 0.017).

| Colony pair | Chi^2^ | d.f. | P- value |
| --- | --- | --- | --- |
| TX1&TX2 | 67.696 | 16 | <0.001 |
| TX1&TX3 | 53.419 | 18 | <0.001 |
| TX2&TX3 | 101.934 | 18 | <0.001 |

Table S7 Hierarchical partitioning of genetic diversity using an analysis of molecular variance (AMOVA) for the overall dataset.

|  | Sum of Squares | Variance component | Percentage | F-stats |
| --- | --- | --- | --- | --- |
| Between populations | 31.308 | 0.250 | 12% | 0.118* |
| Among colonies | 289.938 | 0.576 | 27% | 0.307* |
| Within colonies | 156.500 | 1.304 | 61% | 0.388* |
| Total | 477.746 | 2.131 | 100% |  |

Table S8 Genotypes of *Technomyrmex difficilis* workers in each colony for each of the 12 microsatellite loci

|  |  | Colony |  |  |  |  |  |  |  |  |
| --- | --- | --- | --- | --- | --- | --- | --- | --- | --- | --- |
| Genotypes |  | TX1 | TX2 | TX3 | FL1 | FL2 | FL3 | FL4 | FL5 | FL6 |
| *Ant859* |  |  |  |  |  |  |  |  |  |  |
| 197/197 |  | 22 | 23 | 23 | 7 | 7 | 7 | 7 | 7 | 8 |
| 197/211 |  |  |  |  | 1 | 1 |  |  |  |  |
|  |  |  |  |  |  |  |  |  |  |  |
| *Ant8424* |  |  |  |  |  |  |  |  |  |  |
| 247/247 |  | 4 | 1 | 4 |  | 2 | 3 | 2 |  |  |
| 247/250 |  | 13 | 10 | 8 | 4 |  | 3 |  | 1 | 2 |
| 250/250 |  | 7 | 12 | 12 | 4 | 3 | 2 | 3 | 7 | 6 |
| 250/253 |  |  |  |  |  | 3 |  | 3 |  |  |
|  |  |  |  |  |  |  |  |  |  |  |
| *Ant12200* |  |  |  |  |  |  |  |  |  |  |
| 324/324 |  |  |  |  |  |  |  |  |  | 1 |
| 327/327 |  | 24 | 24 | 24 | 8 | 8 | 7 | 7 | 8 | 4 |
| 327/345 |  |  |  |  |  |  |  |  |  | 3 |
|  |  |  |  |  |  |  |  |  |  |  |
| *LP24* |  |  |  |  |  |  |  |  |  |  |
| 168/168 |  | 12 | 9 | 12 | 7 | 7 | 7 | 7 | 5 | 2 |
| 168/182 |  | 11 | 13 | 9 | 1 | 1 | 1 |  | 1 | 3 |
| 182/182 |  | 1 | 2 | 2 |  |  |  | 1 | 2 | 2 |
|  |  |  |  |  |  |  |  |  |  |  |
| *Ant3653* |  |  |  |  |  |  |  |  |  |  |
| 318/318 |  | 1 |  | 2 | 1 |  | 2 |  |  |  |
| 318/322 |  | 5 |  | 2 |  | 1 |  |  | 3 | 1 |
| 318/324 |  | 5 |  | 5 | 1 |  | 4 |  |  | 2 |
| 320/320 |  |  | 2 |  |  |  |  |  |  |  |
| 320/322 |  | 1 |  | 1 |  |  |  |  |  |  |
| 320/324 |  |  | 7 | 2 |  |  |  |  |  | 1 |
| 320/326 |  |  | 6 |  |  |  |  |  | 1 |  |
| 322/322 |  | 2 |  | 2 | 3 | 2 |  | 1 | 3 | 1 |
| 322/324 |  | 6 |  | 2 | 3 | 2 | 2 | 2 |  |  |
| 324/324 |  | 3 | 2 | 6 |  | 2 |  | 2 |  | 3 |
| 324/326 |  |  | 4 |  |  |  |  | 2 |  |  |
| 326/326 |  |  | 2 |  |  |  |  |  |  |  |
|  |  |  |  |  |  |  |  |  |  |  |
| *Ant1343* |  |  |  |  |  |  |  |  |  |  |
| 208/218 |  |  |  | 1 |  |  |  |  |  |  |
| 218/218 |  | 24 | 24 |  | 3 | 8 | 8 | 8 | 7 | 8 |
| 220/220 |  |  |  | 19 | 5 |  |  |  | 1 |  |
|  |  |  |  |  |  |  |  |  |  |  |
| *Ant7680* |  |  |  |  |  |  |  |  |  |  |
| 287/299 |  | 9 |  | 2 |  |  |  |  |  |  |
| 299/299 |  | 14 | 24 | 21 | 8 | 8 | 8 | 7 | 8 | 6 |
|  |  |  |  |  |  |  |  |  |  |  |
| *Ant20* |  |  |  |  |  |  |  |  |  |  |
| 151/157 |  | 10 |  | 13 |  |  |  |  |  |  |
| 157/157 |  | 13 | 24 | 11 | 8 | 8 | 8 | 7 | 8 | 8 |
|  |  |  |  |  |  |  |  |  |  |  |
| *Ant575* |  |  |  |  |  |  |  |  |  |  |
| 254/268 |  |  |  |  |  |  |  | 2 | 1 |  |
| 256/268 |  | 9 | 8 |  | 1 | 1 |  |  |  |  |
| 268/268 |  | 15 | 16 | 23 | 5 | 7 | 8 | 6 | 7 | 8 |
|  |  |  |  |  |  |  |  |  |  |  |
| *Ant5035* |  |  |  |  |  |  |  |  |  |  |
| 356/356 |  | 24 | 23 | 24 | 4 | 8 | 6 | 2 | 8 | 8 |
| 356/366 |  |  |  |  | 2 |  | 2 | 5 |  |  |
| 366/366 |  |  |  |  |  |  |  | 1 |  |  |
|  |  |  |  |  |  |  |  |  |  |  |
| *L911* |  |  |  |  |  |  |  |  |  |  |
| 262/262 |  |  |  |  | 6 | 7 |  | 7 | 3 | 5 |
| 262/266 |  |  |  |  |  |  |  |  | 1 | 2 |
| 264/264 |  | 12 | 14 | 12 | 2 |  | 5 | 1 | 4 |  |
| 264/266 |  | 7 | 9 | 10 |  |  | 2 |  |  |  |
| 266/266 |  | 4 | 1 | 1 |  | 1 | 1 |  |  | 1 |
|  |  |  |  |  |  |  |  |  |  |  |
| *Ant7249* |  |  |  |  |  |  |  |  |  |  |
| 354/354 |  | 1 |  |  |  |  |  |  |  |  |
| 354/370 |  | 14 | 21 | 8 | 2 | 2 |  | 3 | 1 | 1 |
| 370/370 |  | 9 | 3 | 16 | 6 | 6 | 4 | 4 | 4 | 6 |
